# Design of a 3D High-Definition Map Visualizer for Pose Estimation and Autonomous Navigation in Dynamic Environments

**DOI:** 10.3390/s26041344

**Published:** 2026-02-19

**Authors:** Yunchen Ge, Marcelo Contreras, Neel P. Bhatt, Ehsan Hashemi

**Affiliations:** Department of Mechanical Engineering, University of Alberta, Edmonton, AB T6G 1H9, Canada

**Keywords:** pose estimation, high-definition maps, localization, 3D point cloud data, multimodal data fusion

## Abstract

A high-definition (HD) map development framework providing real-time visualization of multimodal perception data for state estimation, motion planning, and decision-making in autonomous navigation is presented and experimentally validated. The proposed framework integrates synchronized visual and LiDAR data and generates consistent frame transformations to construct accurate and interpretable HD maps suitable for navigation in dynamic environments. In addition, the framework enables flexible customization of essential map elements, including road features and static landmarks, facilitating efficient map generation and visualization. Building upon the developed HD map visualizer, a semantic-aware visual odometry (VO)-based pose estimation module is designed and verified through extensive evaluations and under perceptually degraded conditions. To ensure the reliability of synchronized multimodal data used by downstream perception and pose estimation modules, a sensor health monitoring system is also developed and validated in urban canyon scenarios with intermittent or unavailable global navigation satellite system (GNSS) measurements. Experimental results demonstrate that the proposed HD map visualizer and associated perception modules are transferable for autonomous navigation and can be effectively employed as benchmarking tools for state estimation and motion planning algorithms in autonomous driving.

## 1. Introduction

With the advent of connectivity and sensor networks for multi-modal perception and visual reconstruction of dynamic scenes during autonomous navigation, HD road maps are becoming an essential element for state estimation and motion planning in automated driving systems (ADS) and unmanned ground vehicles (UGVs) in urban/suburban settings. These maps are handy for tasks such as lane detection, trajectory prediction, and motion planning as they (i) provide prior knowledge of traffic scenarios to improve safety [1,2,3,4,5], and (ii) reconstruct the scene for perceptually degraded conditions [6,7]. Thus, an HD map representing the dynamic environment and its accurate visualization are time-vital and safety-critical during autonomous navigation. However, existing solutions are challenged with computational complexity while rendering in real-time due to (i) extensive point cloud data, (ii) inaccurate multi-modal data association before visualization, and (iii) a lack of generalization for learning-based pipelines in various scenes [1,8,9,10,11,12,13].

**Figure 1:**
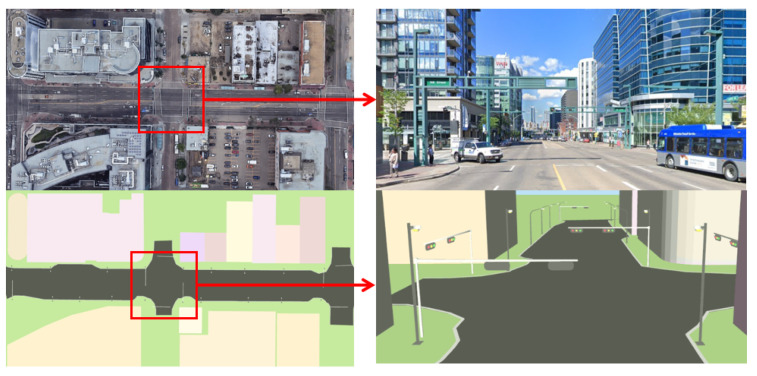
HD map visualization (of a highly dynamic downtown area) augmented with static features including traffic signs/lights and light poles for various intersection geometries. The map is also used for localization purposes.

To this end, we develop a ROS-based HD map generation and visualization framework for autonomous navigation in dynamic environments under perceptually degraded conditions and in GNSS-denied areas (see Figure 1). The developed framework includes road lanes/curbs, sidewalks, and static objects (e.g., traffic lights, traffic signs, light poles, and structures in the vicinity of the drivable region), as well as a vehicle driving simulator with longitudinal speed and yaw rate (in the body frame) as control inputs. The vehicle simulator can also be augmented with an accurate vehicle dynamical model considering tire slips and the effect of combined-slip tire forces on vehicle longitudinal/lateral stability [14,15]. In this regard, a computationally efficient perception system and two sample HD map visualizers are developed using the NODE lab’s perception vehicle and multi-modal data acquisition system.

The accurate road lanes that define road boundaries and curb curvatures/heights are collected by stereo cameras and 128-beam solid-state LiDARs during low-speed navigation and sketched in the visualizer. Various static traffic and environmental features (mentioned above) are also included for traffic rule recognition and the perception module (leveraging these features), which are transferable for mobile robots and UGVs. The framework provides references for the evaluation of Simultaneous Localization and Mapping (SLAM) methods, streaming a compact street representation as inputs to odometry systems (e.g., visual odometry), and generating cost and occupancy maps for motion planning in dynamic scenes [16,17,18]. Furthermore, to ensure the consistency of multimodal data streams during navigation, a ROS-compatible sensor health monitoring system with a programmable indicator is also developed, which is transferable to unmanned ground vehicles (UGVs) and other mobile platforms.

The main contributions of this paper are summarized as follows:

- A fully integrated multimodal perception system is presented for HD map generation in urban autonomous navigation, combining visual and LiDAR sensing with open-source map information to refine and validate feature-of-interest localization, and experimentally evaluated in dense urban environments.
- An HD map-assisted pose estimation framework is developed that uses the proposed HD map visualizer for data augmentation to improve accuracy and robustness in challenging scenarios, together with an automated and computationally efficient visual-LiDAR data association pipeline for objects and landmarks of interest.

The remainder of the paper is organized as follows. Section 2 introduces the multi-modal data acquisition system for the design of the visualizer and preliminaries (i.e., visual-LiDAR data association and transformations). Section 3 presents the process of collecting and editing road features and lanes. Section 4 provides an automated framework for static feature data collections, including refinements for the HD map visualizer, semantic-aware visual odometry, and sensor health monitoring system design. Section 5 reports the results of map quality by correspondence check with the sensor input, state estimations with static map features, and the accuracy of map features.

## 2. Preliminaries and Data Acquisition System

This section introduces the multi-modal data acquisition system used to collect static environmental characteristics to generate the visualizer, then provides the process to develop the road feature inference module, which is transferable for dynamic urban, suburban or highway settings.

**Figure 2:**
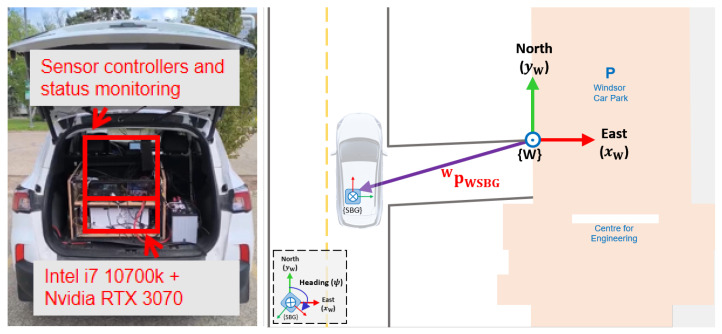
The test vehicle equipped with multimodal visual-LiDAR perception system and GNSS (left); illustration of navigation frames (right).

**Perception and navigation sensors:** The NODE lab’s test vehicle platform (shown in Figure 2) is equipped with stereo vision supporting up to 1920×1200 image resolution at 60 fps integrated in-house, two solid-state LiDARs, one 16-beam LiDAR (to monitor sidewalks and detect pedestrians, scooters, and road curbs), and a GNSS system with the real-time kinematics (RTK) correction and a HFI-A9 IMU. The onboard computer is equipped with a NVIDIA RTX 3070 GPU, and the sampling frequency (for data acquisition) can be from 10 to 50 Hz. The camera intrinsic parameters were calibrated and used for the evaluation of the framework. The GNSS-RTK has the position and orientation accuracies of 2 cm and $0.1^{\circ }$ at 30 s, and is used for HD map generation and validation of the developed semantic-aware visual-odometry system.

**Figure 3:**
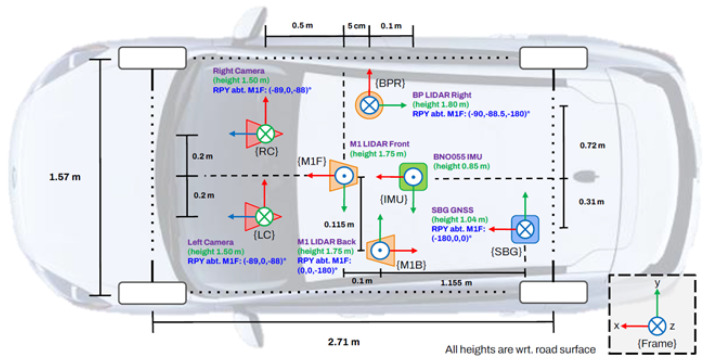
The vehicle test platform and coordinate frames of the multi-modal sensing system.

The frames of the multi-modal sensing system are provided in Figure 3, where the frames of the solid-state LiDARs (with a detection range of 170 m, and a horizontal field of view (FoV) of $115^{\circ }$ for better coverage around the vehicle/robot during autonomous navigation) are shown by {M1F} and {M1B}, and the frame for the side LiDAR is denoted by {BPR}. LiDARs are synchronized with the visual system through the GNSS pulse-per-second (PPS) time synchronization protocol signal. The GNSS frame is shown by {SBG}. The RTK base station is installed near the University of Alberta (UofA) campus for real-time GNSS corrections.

The integrated stereo vision systems allow synchronization by triggering signals. ROS image topics are published at 20 Hz for the visual odometry module and the development of the visualizer.

**Map feature points acquisition:** In the developed perception module and the odometry framework, the estimation of the ego-motion determines the position vector tIJI∈R3 from the frame I to J expressed in the frame I, and the rotation matrix RJI∈SO(3) from the frame J to I. The following homogeneous transformation matrix TJI∈SE(3) is used to represent the pose of the vehicle/robot in a local map frame {W}:

(1) TJI=RJItIJI01.

In order to generate an HD map established with specific objects of interest (e.g., light poles and traffic signs/lights), poses of the GNSS sensor in frame {W} are obtained in low-speed navigation (<10 km h^−1^). The location of landmarks of interest in frame {W} are calculated by

(2) pliW=TSBGWTLSBGpliL

where pliW∈R4 is the position of the $i^{th}$ feature point, TSBGW is the pose transformation from to frame {W} to the GNSS frame {SBG}, TLSBG is the calibrated LiDAR-GNSS extrinsics, and pliW is the position of the corresponding feature point expressed in the LiDAR frame.

The GNSS-RTK data are used as ground truth to calculate the vehicle pose in the local/global coordinate system using the Universal Transverse Mercator (UTM) transformation; an original location is specified where its latitude and longitude are regarded as the origin (x,y) coordinates of the local map frame {W}. The calibration between the LiDAR sensor and the GNSS is also performed to obtain accurate translations/orientations between the GNSS sensor and LiDARs in order to obtain the extrinsic parameters from {SBG} to the LiDAR frame {L}.

The forward conversion of points from the GNSS coordinates (navigation frame) to the frame {W} and its reverse conversion are required to register the collected feature points (from objects) to be included in the HD map. The process converts a point from an ellipsoid to a plane Cartesian coordinate. In this regard, the UTM, which is a common plane coordinate system for global navigation, is utilized for the development of the visualizer and the design of proprioceptive observers in the NODE laboratory for autonomous navigation [19,20,21]. A position in the UTM system is determined by its zone number obtained by Earth’s 60 sector zones (in which the origin of each zone’s Easting and Northing coordinate is the intersection of the equator and the zone’s central meridian) and the north/south hemisphere designator. As summarized in [22], the Krüger series is used for conversion. For a point on the ellipsoid at latitude ϕ and longitude λ, its transverse Mercator Easting and Northing location (x,y) is given by $x=k_{0}A\eta$ and $y=k_{0}A\xi$, where $k_{0}$ is the scale on the central meridian, $A=\frac{a}{1+n}\left(1+\frac{1}{4}n^{2}+\frac{1}{64}n^{4}+\ldots \right)$, and η and ξ are obtained from the Krüger’s series:

(3) $$\begin{array}{cc} \xi & =\xi^{\prime }+\sum_{j=1}^{\infty }\alpha_{j}\sin2j\xi^{\prime }\cosh2j\eta^{\prime },\\ \eta & =\eta^{\prime }+\sum_{j=1}^{\infty }\alpha_{j}\cos2j\xi^{\prime }\sinh2j\eta^{\prime }, \end{array}$$

in which $\xi^{\prime }=g\left(\phi ,\lambda \right)$ and $\eta^{\prime }=\bar{g}\left(\phi ,\lambda \right)$ are provided by (A1) in Appendix A, where the reversed process of transforming a feature point’s transverse Mercator Easting and Northing location to its latitude and longitude is also included. The numerical values of $\alpha_{j}$ and $\beta_{j}$ are provided in [22] up to the 8th terms. The series in the equations are expanded to the 6th terms for the computations of locations in the UTM coordinate by implementation in the UTM module in *Lanelet2* projection package [23] (achieving maximum computation error of 5nm within $35^{\circ }$ of the central meridian [22]).

## 3. Feature Selection and Visualization

The origin of the local map frame {W} is situated on the North Campus of UofA as shown in Figure 2. ROS bags are recorded during low-speed navigation (<10 km h^−1^) in highly dynamic environments (e.g., downtown areas and streets around the campus with several intersections, pedestrian crossings, and bus stations). The recorded bags include the visual inputs (ROS sensor_msgs/CompressedImage) and 3D point clouds (ROS sensor_msgs/PointCloud2) of the environment collected by the perception sensor suite and static and dynamic transformations (ROS tf2_msgs/TFMessage) as in Equation (2). When playing recorded bags in RViz, the topic /clicked_point is utilized to output the location of a point on a given object as visualized in the map environment expressed in frame {W}. With the transformation TSBGW and TLSBG available, the /clicked_point topic computes the position of a selected point expressed in the LiDAR frame in the visualizer to that expressed in frame {W}.

The feature points for road lanes and curbs are selected from LiDAR point clouds with available corresponding RGB images. By selecting the frame {W} as a fixed frame and tracking the frame {SBG}, the related point clouds close to the vehicle and on-road lanes/curbs are identified and selected, and their positions pliW are published. Utilizing this information, the perception module for the developed visualizer uses the *Lanelet2* format for handling map data and the *JOSM* editor, which utilizes *OpenStreetMap* [24], map data to fine-tune specific labels and save HD maps.

**Figure 4:**
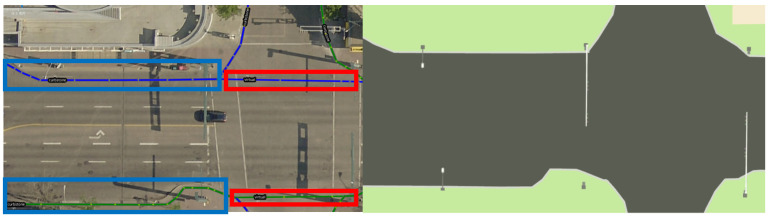
Traffic elements and road lanes in the visualizer for the *JOSM* editor (blue: curbstone lanes, red: virtual lanes) at a downtown intersection.

The UTM module in the *Lanelet2* package is utilized to convert locations collected with bags in RViz in frame {W} to global GNSS coordinates by specifying the map frame origin’s latitude and longitude in GNSS coordinates (as provided in the previous section). The converted discrete locations are written into a XML map file that is parsed by *JOSM* to accurately localize the selected scatter points of road lanes visible in the top view of satellite images in the editor. Then, the road lanes are sketched in the editor by following the format requirements of *Lanelet2*. Figure 4 depicts examples of intersections in *JOSM* and in the developed HD map visualizer with three major road lane types. The following items summarize the format requirements of the three types in the *JOSM* editor:

1. Curbstone lanes: two pairwise lines of nodes included in one road relation with left and right lanes and they refer to the road curbs (i.e., spatial constraints).
2. Virtual lanes: that maintain the roads’ continuity.
3. Pedestrian lanes: virtual lanes which allow pedestrians to pass a section.

**Figure 5:**
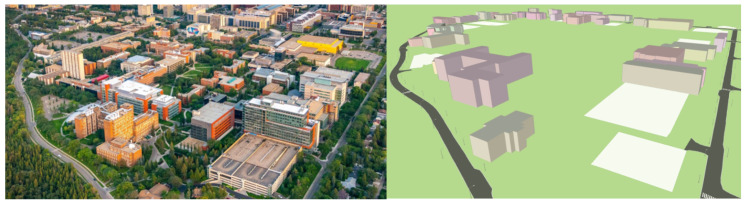
Aerial view of the UofA North campus loop ((**left**) copyright by the University of Alberta); and the developed HD map visualization (**right**).

The map loader, map visualizer, and ROS interface modules in *Lanelet2* handle the positioning and visualization of the road lanes in the edited XML map file. In this regard, (i) locations of pairwise road lanes are parsed from the XML file and converted back to their corresponding locations in the local map frame using Equation (3), and (ii) the visualization module publishes a visualization_msgs/MarkerArray ROS topic so that the road lanes can be displayed in RViz. A full aerial view of the developed HD map for a university campus setting is shown in Figure 5.

## 4. Augmented Feature Appearance and Pose Estimation

The existing *Lanelet2* visualization is augmented in this paper so that the main features in the HD map can be one-to-one scaled. This is useful for tasks such as comparing the results of static object perception and motion planning, as accurate results can be validated directly through the HD map visualizer. This is done by creating custom models matching actual object appearances and sizes in Blender software [25]. The objects of interest are imported into Blender from 3D model files and modified with custom colors, and their coordinate frames are set to a desired location.

The processed models are exported as mesh files, identifying the model color, size, and orientation when published as a ROS visualization_msgs/MarkerArray message type topic in RViz software. Custom labeling of features in the *JOSM* editor and ROS nodes to parse feature locations and orientations are created after the feature points are collected. For collecting the points of static objects, we differentiate the processes for a subset of the objects detected in a bounding box, which is then collected automatically. The data collection process for feature points using associations of object detection results (of bounding boxes from RGB images and LiDAR point clouds) is discussed in the next section.

The main static features included in the proposed perception and visualization framework are accurate curb locations, light poles, traffic signs/lights, buildings, pedestrian crossings, and parking areas. The following summarizes the format for these elements in *JOSM*: (i) Stop signs, lines of nodes (for all the stop signs at an intersection) included in one regulatory element relation with all the short lines labeled as refers; (ii) Traffic lights, a line of nodes called as traffic_light_ref which is connected to traffic_light_box and traffic_light_pole nodes; (iii) Parking areas, a multi-polygon relation with enclosed lines of outer nodes and a parking sub-type; (iv) Buildings, a multi-polygon relation with enclosed lines of outer nodes and a building sub-type; and (v) Light poles, a line of light_pole_ref nodes labeled as with front and back nodes.

The MarkerArray type ROS topic supports batch visualization of objects with different appearances from different mesh files. After parsing the locations and orientations of objects of the same type (but different appearances) and publishing the object poses in frame {W}, a ROS node differentiates the published object appearances and assigns the correct mesh file to each object at its designated pose in the map frame. Then, it publishes a MarkerArray-type ROS topic for the object class.

A generated mesh file is the source file for a ROS URDF model (for the vehicle/robot). We add a navigation frame to the URDF model, {SBG} frame for ground truth, and left camera frame {LC} for visual navigation so that the vehicle/robot mesh is attached to this frame during autonomous navigation.

### 4.1. Automated Visual-LiDAR Feature Collection

In order to automate the data collection of several types of landmarks from RGB images and include the multi-dimensional landmark features required for the visual navigation module, an algorithm is developed in this section to associate the detected object in a bounding box $B_{i}$ to its corresponding point cloud pliboxL in the LiDAR frame and, subsequently, obtain the object’s distance from the associated point cloud. Consider a time-synchronized RGB image input and LiDAR point clouds of landmark $l_{i}$, as well as positions and dimensions (i.e., width and height $B_{i}^{w},B_{i}^{h}$) of bounding boxes containing objects of interest. The projected 2D position zliC of 3D LiDAR points pliL onto the image is given by

**Figure 6:**
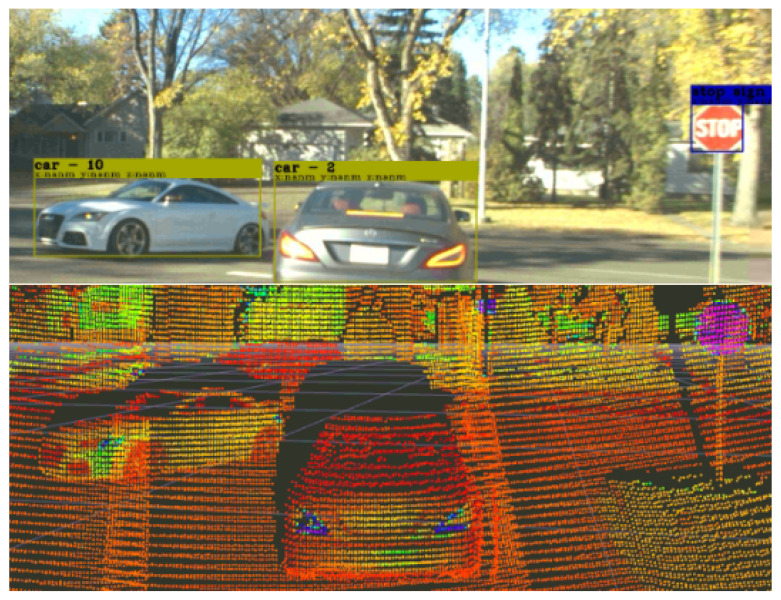
Depth measurement and extraction of landmark of interest (e.g., stop/traffic signs) through monocular vision and LiDAR point cloud data association.

(4) zliC=KTLCpliL,

where K is the camera intrinsic and TLC is the calibrated extrinsic transformation between LiDAR and camera. As shown in Figure 6, a subset of points pliboxL, which lies on the interior $I_{B_{i}}$ of the bounding box when projected onto the RGB image, is extracted by comparing all zliC with the 2D positions that the bounding boxes occupy.

The points in the zliC cluster and the distance of the corresponding object of interest can be calculated from the mean of clustering. To this end, (i) the points in pliboxL are divided into different distance ranges with discrete distance intervals (e.g., 0.2 m) and sorted to find the distance interval into which the largest number of points fall, (ii) a specific value within the obtained distance interval (i.e., center of the interval) is selected, and (iii) the mean of the object distance is computed by averaging the *M* points in pliboxL which have the distances within a threshold percentage of the selected value (e.g., ±5%). This process is automated, and the vehicle-to-landmark data points are collected by landmark’s location in the LiDAR frame. Then, they are expressed in the local map frame using (2). A summarized version of this process and object distance estimation is provided in Algorithm 1.

**Algorithm 1:** Visual-LiDAR data association

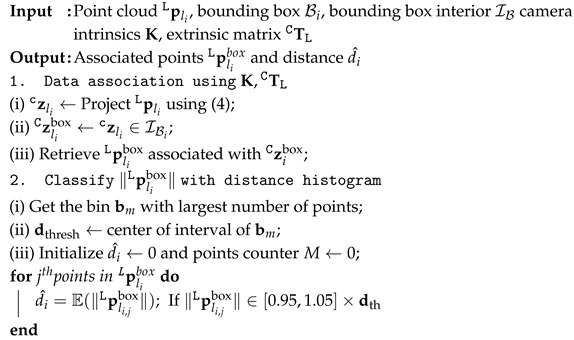



The remaining landmarks (i.e., buildings, bridges, public transportation stops) are collected manually based on semantics and distribution of their point clouds in the LiDAR frame {L} (e.g., L-shape forms in the LiDAR frame shown in the next section) It should also be noted that the integration and fusion of infrastructure-based multimodal sensing [26] with onboard perception systems of automated driving platforms will (i) enhance the scalability, large landmarks’ reconstruction accuracy, and automation of HD map generation, and (ii) enable real-time dynamic update of environmental data. Such an extended sensing framework (as the future work) is expected to support tighter coupling between the proposed visual odometry-based pose estimation and the HD map visualizer, enabling more comprehensive digital twin representations of complex urban environments under diverse operational and perceptual conditions.

### 4.2. Pose Estimation Leveraging HD Map Visualizer Data

Static features are crucial in visual/LiDAR-based state observer design since they are robustly trackable in different frames and are easily integrated into the developed HD map for long-term data association. The latter helps to compute finer representations of the surroundings for structure-from-motion approaches [27] along the map to achieve consistency beyond consecutive frames. In this regard, and based on our previous works [21,28], a semantic-aware visual odometry (VO) is designed and integrated with the visualizer developed for autonomous navigation in perceptually degraded conditions to enhance safety and reduce initialization error where necessary.

**Figure 7:**
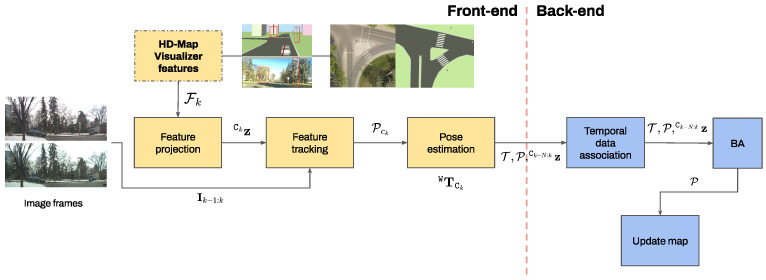
Integration of feature-based VO which retrieves the visualizer static features to track them in consecutive views and maintains a local map for longer horizon tracking. The framework is general enough to adopt data association techniques based on feature matching or in optical flow.

Figure 7 depicts the integrated architecture of the proposed visualizer and the VO to estimate the ego-vehicle pose. In the proposed integrated framework, (i) the visualizer features are convenient additions to external pose and speed estimation modules, and (ii) their information is generalizable for scene reconstruction to various visual navigation scenarios for UGVs and automated driving in urban settings.

First, the HD map filters the features corresponding solely belonging to static classes and transforms them from LiDAR frame {L} to camera frame {LC}. Then, the VO module projects the HD map features pCk to the image plane zCk with the pinhole camera model assuming that rectification has already been applied. Feature tracklets are generated by LKT [29] between two consecutive frames k−1 to *k*. To discard the outlier tracklets, we apply a backward optical flow verification and discard any tracklets with more than 0.5 px error. Since the HD map provides the corresponding 3D points, we utilize a 3D-2D pose correspondence (e.g., PnP [30]) with a RANSAC outlier scheme to estimate the pose TCkW. Features and poses are appended in a temporal window, and features are associated across distant frames based on binary descriptor matching (i.e., Hamming distance).

To refine the previous estimations, bundle adjustment (BA) optimization is executed to optimize the last *N* camera poses T (a set of TCkW transformations) and the 3D points P with their respective image features $\hat{z}$ from landmarks, aiming for local pose consistency over a temporal horizon:

(5) {T,P}=argmin∑ck∈T∑li∈Pckρ(E(ck,li)),E(ck,li)=||z^liCk−π(WT^Ck⊟pliW)||Σ2

where T^CkW is the $k^{th}$ camera pose with respect to frame {W}, pliW is the 3D point of the $i^{th}$ landmark seen in the $k^{th}$ frame, z^liCk is the image feature from the $i^{th}$ point seen in the $k^{th}$ frame, ⊟ is the retraction operator defined for SE(3) manifold, $P_{c_{k}}$ are the landmarks associated with the features seen in the $k^{th}$ camera frame, ρ(·) is a robust loss function for outlier removal, π(·) is the pinhole camera projection, and Σ is the information matrix (in the Mahalanobis distance sense). In order to execute BA in (5), data associations must be known prior.

Algorithm 2 elaborates on the elements of the proposed framework, including feature tracking, depth estimation, and bundle adjustment, to update the camera poses T and the map composed of 3D points P.
**Algorithm 2:** Integrated visualizer-VO
**Input**   **:**Camera intrinsics K, visualizer features $F_{k}$, and RGB image $I_{k}$, $N_{c}$ window horizon
**Output:** Pose TCkW, 3D points of P
**while** k≥0 **do**    1. Feature detection/tracking;  Retrieve image projections zCk and 3D-points pCk from $F_{k}$   **if** k≥0 **then**
      $P_{c_{k}}\leftarrow$ FeatureTracking(zCk−1:k,Ik−1:k);
   2. Pose estimation(PnP) [31];
   TCkCk−1← Pose estimation using pCk−1, zCk;
   TCkW=TCk−1WTCkCk−1;
   3. Moving horizon optimization;
   $T\leftarrow T\cup^{W}T_{C_{k}}$;
   $P\leftarrow P\cup P_{c_{k}}$;
     **if** $\mathrm{card}\left(T\right)=N_{c}$ **then**
     P← AssociateData($T,P{,}^{C_{k-N:k}}z$);
    $\left\{T,P\right\}\leftarrow \mathrm{BA}\left(T,P{,}^{C_{k-N:k}}z\right)$ by (5);
    T={⌀},P={⌀}
     **end**
    **end**
  **end**


### 4.3. Sensor Status Monitoring System

**Figure 8:**
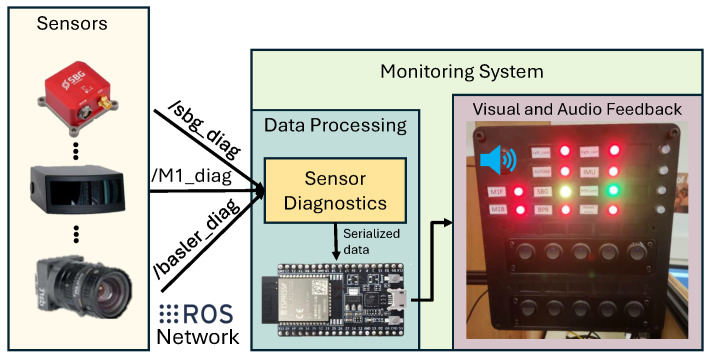
Sensor status monitoring system integrated with the perception module for autonomous navigation.

The consistent data stream of GNSS-RTK, cameras, and solid-state LiDAR sensors is required to generate the HD map, state estimation, and motion planning/control modules for the NODE lab’s test vehicle. A status monitoring system has also been developed to detect information dropout and ensure the consistency of the above-mentioned multi-modal sensor data during navigation in highly dynamic environments. The monitoring system comprises an ESP32 microcontroller module, an LED strip indicator, and a low-power buzzer. For software components, rosserial_python and the ROS components for the microcontroller are used. A ROS node is set up to periodically check if the ROS topic received from the multi-modal sensor array is at a reliable state (using consistency filters) and to publish an unsigned 32-bit number ROS topic. The serial_node script within the rosserial_python [32] module bridges the computer and ESP32 so that the microcontroller subscribes to any ROS topics in the same way (as other ROS nodes on the computer). Using the predefined protocol based on the unsigned number, ESP32 periodically obtains the status of each sensor by bit-wise operations (which parses one sensor’s corresponding bit information for its actual status); then, LED colors are assigned to indicate the status accompanied by the buzzer signal. Figure 8 illustrates the monitoring system elements/architecture.

The monitoring system has a high refresh rate, a visual indicator of the consistency of the onboard multi-modal sensors data stream, and wireless communication with a base station for remote monitoring and takeover in safety-critical situations.

## 5. Experimental Results and Discussion

In this section, HD map examples augmented with main static features for localization and vehicle state estimation in highly dynamic downtown and hybrid campus–residential environments are provided where essential features (e.g., accurate curb locations/heights, traffic signs, light poles, and pedestrian crossing) are generated for autonomous navigation.

### 5.1. HD Map and Visual-LiDAR Feature Collection

**Figure 9:**
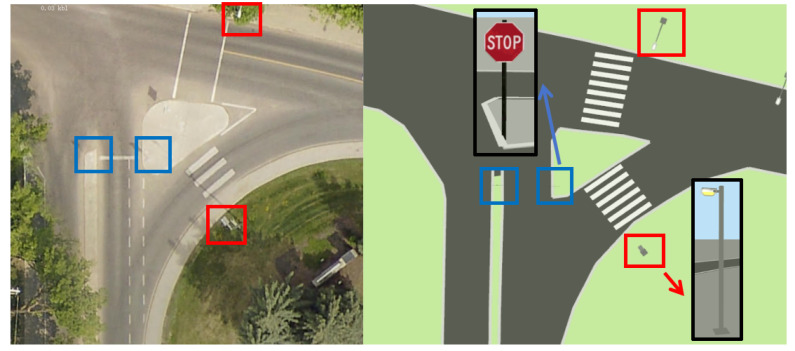
Landmarks and pedestrian lane type in the visualizer for visual-based estimation in dynamic scenes, and reliable initialization in visual odometry; traffic signs and poles are labeled with blue and red boxes, respectively.

Figure 9 presents a close view of a representative region within the developed HD map visualizer, augmented with traffic-related features that act as stable landmarks for localization in dynamic scenes with multiple moving objects. Such semantically meaningful features support spatial consistency and improved visual odometry accuracy in dynamic urban settings using the HD map visualizer. Figure 1 shows a partial HD map of public roads in a highly dynamic downtown area of Edmonton, AB, demonstrating the capability of the proposed framework to capture detailed road geometry and surrounding static landmarks. Such an interpretable HD map supports reliable feature-based localization and navigation in dense urban environments.

**Figure 10:**
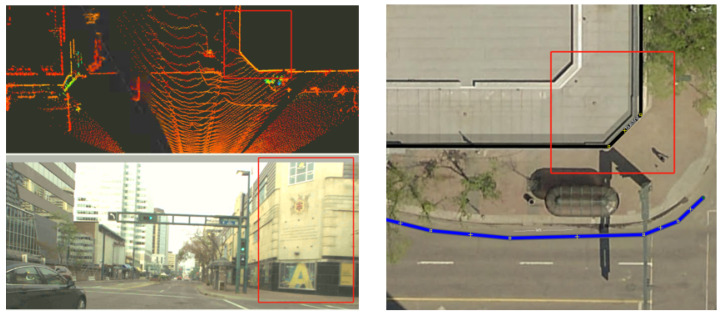
Location refinement of landmarks (buildings and road curbs) in *JOSM* through processed point clouds (**left**); top view (**right**).

**Figure 11:**
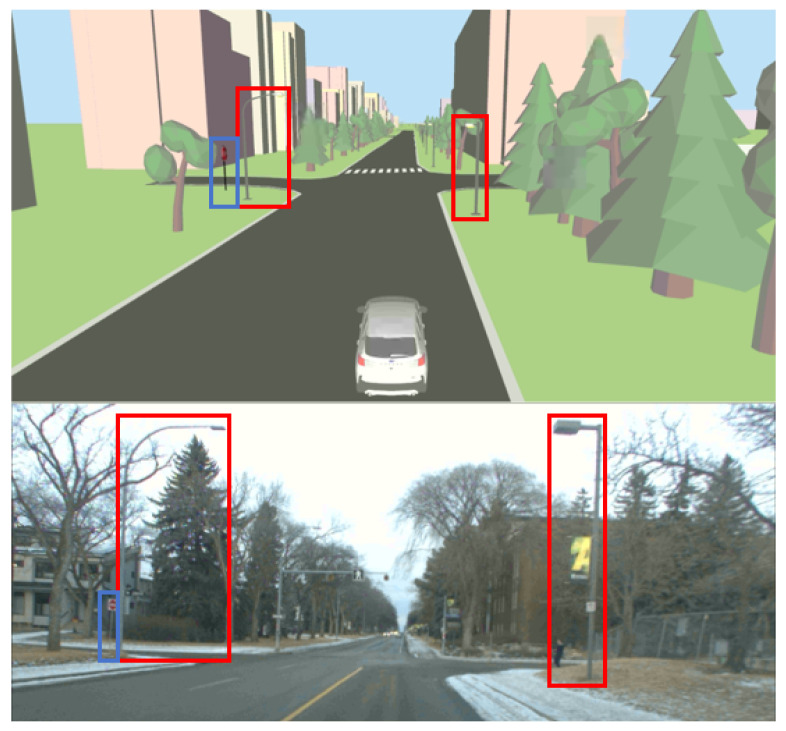
Refined landmarks for visual odometry during autonomous navigation: HD map representation with accurate customized landmark positions (**top**); unstructured environment with vegetation (**bottom**).

Figure 10 illustrates the output of the automated visual–LiDAR feature collection process for another landmark (building stricture). The reconstructed L-shaped point cloud at an intersection, together with the associated road curb, demonstrates the capability of the HD map visualizer to capture key structural features relevant for localization. This hybrid automated–manual selection of the remaining landmarks addresses two major cases prone to point selection errors: (i) scenarios in which the vehicle approaches the landmark, and (ii) partial occlusion of the landmark’s principal planes due to dense vegetation (as shown in Figure 11), large vehicles/trucks, or public transportation vehicles (when multiple points on the landmarks are collected and published with /clicked_point topic in RViz). These results indicate that the proposed approach enables reliable feature extraction and map augmentation even under partial visibility conditions, supporting robust landmark-based localization.

**Figure 12:**
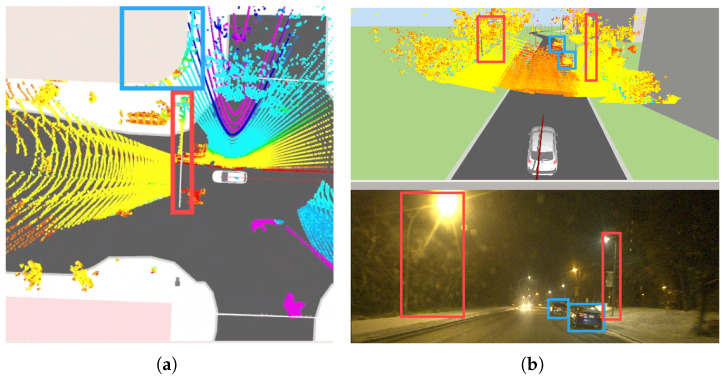
(**a**) Correspondence between landmark locations and LiDAR point cloud measurements for navigation in dynamic environments (e.g., buildings marked in blue and traffic lights marked in red); (**b**) vehicle perception using LiDAR point cloud data in the map visualizer under degraded perception conditions (blue: vehicles, red: light poles).

The developed HD map visualizer is compatible with 3D spatial data from multiple sensors available in RViz, including LiDAR and radar point clouds, enabling integrated visualization for classification, localization, and scene reconstruction tasks. Accurate correspondences between static landmarks in the visualizer and LiDAR measurements (from a top-view autonomous navigation scenario in dynamic environments) are demonstrated in Figure 12a, maintaining spatial consistency between the mapped features and real-time sensor observations and supporting the evaluation of semantic-aware visual odometry and landmark-based localization in the digital twin environment. Figure 12b presents the output of the multimodal perception module using LiDAR data under perceptually degraded, low-light conditions, and confirms that the visualizer preserves feature correspondence and interpretability even when visual sensing is limited for navigation in challenging environments.

### 5.2. Semantic-Aware Visual Odometry

**Figure 13:**
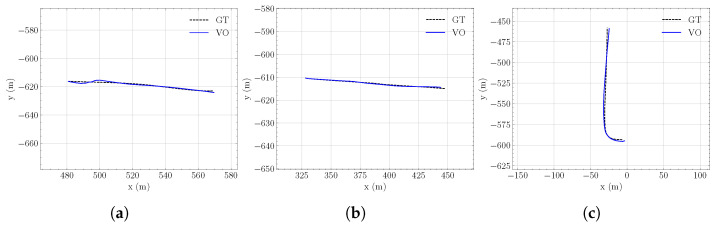
Estimated trajectories by the visual odometry pipeline using HD map features ((**a**): sequence 1, (**b**): sequence 2, and (**c**): sequence 3). Ground truth (GT) trajectories were collected from GNSS.

**Figure 14:**
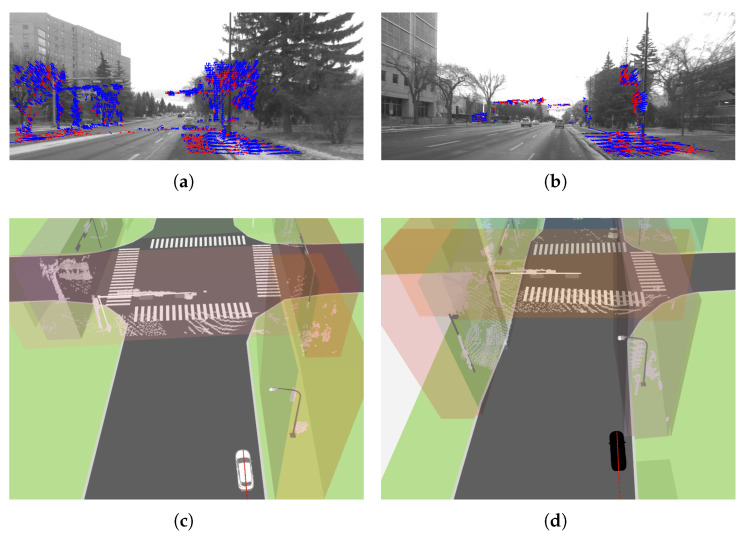
(**a**,**b**) illustrate motion tracking results, where blue tracks denote inliers and red tracks denote outliers, demonstrating that the majority of inliers are consistent with the vehicle’s ego-motion. (**c**,**d**) show the 3D bounding boxes used to filter LiDAR scans at static landmarks over the developed HD map framework. The 3D bounding boxes are dilated to prevent overly sparse feature selection.

The integration of static HD map features (generated by the proposed framework) with the VO module demonstrates improved pose estimation accuracy, as quantified by the Average Pose Error (APE) for both translation and rotation in Table 1. Across three dynamic sequences collected in the UofA North Campus ring, the method achieves absolute translation errors below 2.5 m, reaching sub-meter accuracy for Seq. 1, while consistently maintaining orientation errors below 0.1 rad. Qualitative comparisons between the estimated trajectories and GNSS ground truth, shown in Figure 13, further confirm close alignment and consistency of the estimated paths. The performance of the static feature-tracking module in urban navigation scenarios is illustrated in Figure 14. The HD map provides a dense set of trackable features that can be effectively processed using the LKT tracker to capture the camera’s dominant optical flow associated with ego-motion, with only a small proportion of features remaining untracked. These results demonstrate accurate localization and robust tracking using features in the visualizer, supporting consistent map-referenced motion estimation. Furthermore, the proposed HD map visualizer plays an important role as a tool for data augmentation and scenario generation, enabling controlled evaluation of visual odometry systems under diverse environmental/feature configurations.

Analysis of accuracy of map features: Absolute and relative accuracy are used to evaluate map features generated by the proposed framework. The mean values of the two metrics for the overall map features are absolute accuracy <15 cm and relative accuracy 25 cm for road lanes, and absolute accuracy <17 cm for static landmarks using the designed visual-LiDAR data association framework and the state observer. Absolute and relative accuracy metrics are used for the road lanes for which overall continuity is essential. Then, discrete static landmarks are evaluated using absolute accuracy. The precision of the proposed HD map generation framework and GNSS for several datasets in highly dynamic downtown areas and urban canyons are also compared in Table 2 with other commercially available HD maps (summarized in [33]).

**Figure 15:**
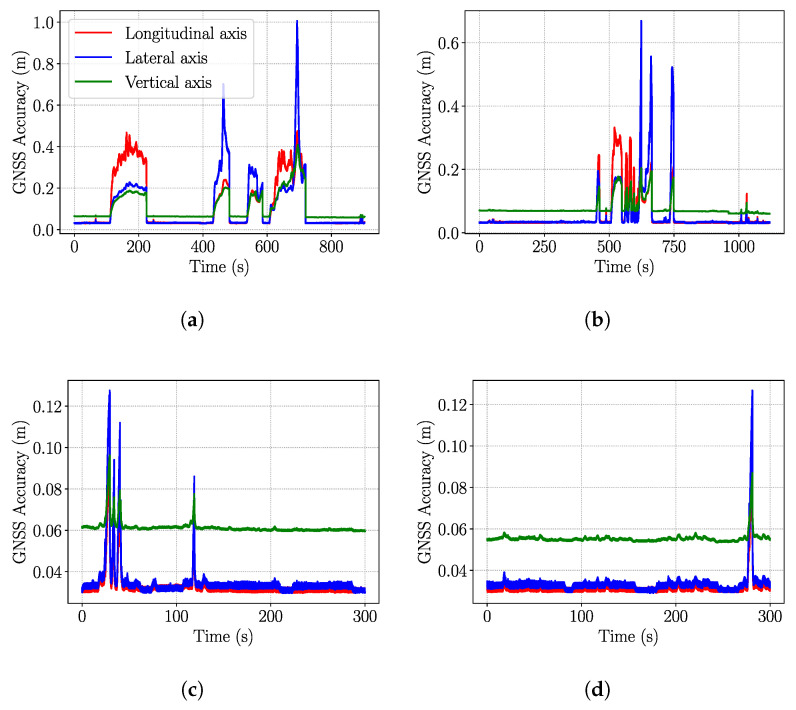
GNSS accuracy in highly dynamic environments and urban canyons with loss of reception: (**a**) UofA campus with intersections, (**b**) full visual-based navigation around the university campus’ ring road, (**c**) navigation in downtown areas, and (**d**) navigation in downtown with loss of reception (due to urban canyons.

Furthermore, Figure 15 presents the accuracy of the GNSS for some of datasets gathered in dynamic downtown areas and UofA’s North campus.

Supplementary Materials, including a recorded video of the HD map visualizer (with the multimodal perception system) and the visual odometry framework is provided with this submission too. The video also show the performance of the proposed visual odometry integrated with the HD map visualizer in real-time.

## 6. Conclusions

A real-time HD map visualizer and a perception system for autonomous navigation and state estimation in dynamic urban settings were developed. Modules for semantic inference from surroundings, necessary frame transformations and software packages, data collection, and multi-modal data association procedures were also presented. Furthermore, an integrated model of the visualizer and a visual odometry framework were also provided for accurate pose estimation. As confirmed by several experimental tests in various environments, the developed visualizer and perception module is transferable for autonomous navigation in dynamic urban/suburban or highway settings to enhance state estimation’s accuracy and reliability and to evaluate decision-making and motion planning strategies. To ensure the reliability of multi-modal data acquisition, a real-time monitoring system was also designed to evaluate the consistency and operation of individual sensors in real time under normal and perceptually degraded conditions.

The visualizer provides sufficient information as a digital twin for evaluation decision-making and path-planning algorithms in a safe, reproducible, and controllable environment. Future research avenues and system development include: (i) more automated collected map features based on semantic information; (ii) higher levels of automation in large-scale scene construction through communication with infrastructure sensors for fine reconstruction of large landmarks (e.g., buildings’ facades) to improve the accuracy of the HD-map visualizer in dense urban settings; and (iii) adding vehicle dynamics models with combined-slip tire forces to evaluate decision-making during higher-speed driving at lateral/longitudinal stability limits considering tire force capacities. The upcoming work of the authors focuses on increasing the level of automation in large-scale scene reconstruction through the integration of multimodal sensors deployed on intelligent mobility infrastructures (e.g., roadside cameras, LiDAR units) with onboard perception systems of automated driving platforms. This integration is expected to enhance the scalability and robustness of the proposed visual odometry-based pose estimation and HD map visualization framework, supporting digital twin applications in complex and dense urban environments. In this regard, sensor network systems are being developed for installation on infrastructures for dynamic configurations and updating of the HD map.

## Figures and Tables

**Table 1:** Quantitative results of Absolute Pose Error (APE) in translation (T) and heading (R) for dynamic scenes in North Campus UofA.

Seq.	Evaluation Metric	
	${\mathrm{APE}}_{T}$ **[m]**	${\mathrm{APE}}_{R}$ **[rad]**
1	0.752	0.051
2	2.392	0.095
3	2.432	0.087

**Table 2:** Map accuracy comparison.

Map Products	Absolute Accuracy	Relative Accuracy
HD Map with RoadDNA [34]	<100 cm	15 cm
Sanborn HD Map [35]	7–10 cm	-
Apollo HD Map [36]	-	10–20 cm
NavInfo OneMap [37]	-	10–20 cm
Our HD map visualizer	<18 cm	17 cm

## Data Availability

The original contributions presented in this study are included in the article. Further inquiries can be directed to the corresponding authors.

## Supplementary Materials

The following supporting information can be downloaded at: https://www.mdpi.com/article/10.3390/s26041344/s1.

## Appendix A. Navigation Model Parameters

The parameters associated with Krüger’s method used in the transformation process, described in Section 2 for augmenting the HD map with newly observed landmarks and features, are summarized as follows. Let *a* denote the equatorial radius of the reference ellipsoid and *b* its polar semi-axis. The flattening *f* and eccentricity *e* are defined as f=(a−b)/a and $e=\sqrt{f(2-f)}$, respectively, while the third flattening is given by n=(a−b)(a+b)=f/(2−f). The parameters $\xi^{\prime },\eta^{\prime }$ introduced in the same subsection are defined as $\xi^{\prime }=\tan^{-1}\left(\tau^{\prime }/\cos\lambda \right)$ and $\eta^{\prime }=\sinh^{-1}\left(\sin\lambda /\sqrt{\tau^{\prime 2}+\cos^{2}\lambda }\right)$, where $\tau^{\prime }=\tau \sqrt{1+\sigma^{2}}-\sigma \sqrt{1+\tau^{2}}$, τ=tanϕ, and $\sigma =\sinh(e\tanh^{-1}\left(e\tau \sqrt{1+\tau^{2}}\right))$. Consequently, the inverse transformation is expressed as $\eta =x/(k_{0}A)$ and $\xi =y/(k_{0}A)$, where $k_{0}$ and *A* are defined accordingly. Then, $\xi^{\prime }$ and $\eta^{\prime }$ are computed by

(A1) $$\xi^{\prime }=\xi -\sum_{j=1}^{\infty }\beta_{j}\sin2j\xi \cosh2j\eta ,$$

(A2) $$\eta^{\prime }=\eta -\sum_{j=1}^{\infty }\beta_{j}\cos2j\xi \sinh2j\eta$$

which yields the intermediate variables $\tau^{\prime }=\sin\xi^{\prime }/\sqrt{\sinh^{2}\eta^{\prime }+\cos^{2}\xi^{\prime }}$ and $\lambda =\tan^{-1}\left(\sinh\eta^{\prime }/\cos\xi^{\prime }\right)$. The parameter τ required for the latitude calculation $\phi =\tan^{-1}\tau$ is subsequently recovered from $\tau^{\prime }$ using an iterative Newton–Raphson procedure:

(A3) $$\tau_{i}=\left\{\begin{array}{cc} \tau_{i}^{\prime }, & \mathrm{for}\;i=0,\\ \tau_{i-1}+\delta \tau_{i-1}, & \mathrm{otherwise}, \end{array}\right.$$

where $\tau_{i}^{\prime }=\tau_{i}\sqrt{1+\sigma_{i}^{2}}-\sigma_{i}\sqrt{1+\tau_{i}^{2}}$ and the Newton update $\delta \tau_{i}$ is given by $\delta \tau_{i}=\frac{\tau^{\prime }-\tau_{i}^{\prime }}{\sqrt{1+{\tau_{i}^{\prime }}^{2}}}\frac{1+\left(1-e^{2}\right){\tau_{i}}^{2}}{\left(1-e^{2}\right)\sqrt{1+\tau_{i}^{2}}}$.
